# Association of participation in the Northern Finland Birth Cohort 1986 with mental disorders and suicidal behaviour

**DOI:** 10.4178/epih.e2022005

**Published:** 2022-01-03

**Authors:** Martta Kerkelä, Mika Gissler, Juha Veijola

**Affiliations:** 1 Research Unit of Clinical Neuroscience, University of Oulu, Oulu, Finland; 2 Finnish Institute for Health and Welfare, Helsinki, Finland; 3 Research Centre for Child Psychiatry, University of Turku, Turku, Finland; 4 Academic Primary Health Care Centre, Region Stockholm, Stockholm, Sweden; 5 Department of Molecular Medicine and Surgery, Karolinska Institute, Stockholm, Sweden; 6 Department of Psychiatry, University Hospital of Oulu, Oulu, Finland; 7 Medical Research Center, University Hospital and University of Oulu, Oulu, Finland

**Keywords:** Psychiatry, Suicide, Follow-up studies, Finland

## Abstract

**OBJECTIVES:**

In prospective follow-up studies, participants are normally contacted during the follow-up period. Even though the idea is not to intervene, the studies conducted during follow-up may affect the target population. Our hypotheses were that participation in the prospective Northern Finland Birth Cohort 1986 study (NFBC 1986) increased the use of mental health services and reduced suicidal behaviour due to participation in follow-up studies.

**METHODS:**

The NFBC 1986 study covered people with an expected date of birth between July 1985 and June 1986 in northern Finland (n=9,396). The participants of the NFBC 1986 were followed since the antenatal period with follow-ups including clinical examinations. The comparison cohort comprised people born in the same area in 1987 (n=8,959), who were not contacted. Registry data on psychiatric treatment, suicide attempts, and suicides were available. Crude risk ratios (RRs) and adjusted (for marital status and education) Mantel-Haenszel RRs were reported.

**RESULTS:**

No increase in mental disorders were found in NFBC 1986 compared to comparison cohort. In the crude RR analysis of female participants, a lower risk for suicide attempts was found (RR, 0.67; 95% confidence interval, 0.49 to 0.92; p=0.011).

**CONCLUSIONS:**

The results did not support our first hypothesis regarding the increased use of mental health services in the NFBC 1986 cohort. However, our second hypothesis gained some support as female participants of the NFBC 1986 had a lower risk of suicide attempts, although it was not due to a higher number of participants receiving psychiatric treatment.

## INTRODUCTION

Prospective longitudinal follow-up studies are popular study designs, in which longitudinal exposure-outcome assessments are studied. The data collection procedures include clinical examinations, questionnaires, tests, interviews, or linkage to existing data [1]. The British birth cohort of the Avon Longitudinal Study of Parents and Children originally consisted of pregnant female in the greater Bristol area between April 1991 and December 1992 (n= 14,500). The mothers, fathers, children, and grandchildren have been followed since with questionnaires and clinical examinations [2]. The Danish National Birth Cohort recruited 100,000 female in early pregnancy, and initial data collection was conducted through telephone interviews with female during pregnancy (1996-2002). The mothers and children then participated in multiple interviews and questionnaires [3]. There are also many newer longitudinal follow-up birth cohorts; for example, the Korean Ewha Birth and Growth Study recruited 891 pregnant female in 2001 in Seoul. The mothers completed multiple follow-up questionnaires and the children had check-up examinations during childhood and adolescence [4]. In the largest Canadian general population-based longitudinal study, the Canadian Healthy Infant Longitudinal Development study, 3,624 pregnant female, most of their partners, and 3,542 eligible offspring were originally recruited in 2008-2012. The mothers, partners, and children have since been followed-up with questionnaires, clinical examinations, and blood samples [5]. In all the above-mentioned birth cohorts, multiple research questions have been studied in different fields. The follow-up procedures in longitudinal birth cohort studies raise the question of whether the intensive follow-up could have affected the study population in any way. Even though the idea is not to intervene in order to obtain a representative sample of the population, the studies conducted during follow-up may affect the target population in various ways.

The large Finnish population-based Uusikaupunkiand Kemijärvi (UKKI) study explored the stability and changes of mental health in the adult Finnish population during 1970-1987. The study sample was followed for 16 years using questionnaires, interviews, and health care registers. The association between participation in the UKKI-study and mental healthcare use was examined by comparing the participants to a matched control group. The UKKI study participants were found to have used mental healthcare services more often than the control group during the follow-up. This result may be regarded, at least partly, as an effect of the follow-up in the UKKI study [6].

There is evidence that psychological [7] and social interventions [8] can be effective in preventing self-injurious thoughts and behaviours, but less intense mobile-based and Internet-based interventions have also proven to be effective [9]. There are also multiple successful suicide prevention programs, such as the national suicide prevention program in Finland, with a reduction of 9% in the incidence of suicide achieved over the entire duration of the project. Its actions included taking care of high-risk people, prevention of marginalisation among young male, and providing an enchanting educational and cultural atmosphere [10].

We studied the associations of suicidal behaviour and the use of mental health services with original participation in a longitudinal study that started prospectively in the antenatal period, namely the Northern Finland Birth Cohort 1986 (NFBC 1986). The purpose of this study was to examine whether membership in the NFBC 1986 had any effect on the use of mental health services or suicidal behaviour. Our hypotheses were that participation in the NFBC 1986 cohort would increase the use of mental health services and reduce suicidal behaviour due to participation in the follow-up studies.

## MATERIALS AND METHODS

### Cohorts

The NFBC 1986 study covered people with an expected date of birth between July 1, 1985 and June 30, 1986 in the 2 northernmost former provinces in Finland, Oulu and Lapland. The cohort comprised 9,362 mothers and 9,479 children. The NFBC 1986 is a longitudinal and prospective birth cohort with several follow-ups [11]. In the current study, only register data of the members of NFBC 1986 were used, and not the data gathered in follow-up studies of the NFBC 1986.

The comparison cohort comprised all people born in the Lapland and Oulu provinces in 1987. The comparison cohort is a subsample from the Finnish Birth Cohort 1987 (FBC 1987). The FBC 1987 study followed all children who survived the perinatal period (were alive at 7 days from delivery) born in Finland in 1987. The FBC 1987 was a register-based study and all the collected data were obtained from several registrars [12]. The members were never contacted during follow-up. Information on the place of birth was obtained from the Medical Birth Register, which is currently maintained by Finnish Institute for Health and Welfare (THL).

### Follow-ups in the Northern Finland Birth Cohort 1986

The first NFBC 1986 follow-up was conducted during the mother’s pregnancy. The follow-up included 3 questionnaires for the mother. Clinical data from antenatal clinics were also collected. The questionnaire included questions about mother’s background, smoking and alcohol use, general health, fatigue during pregnancy, and delivery (Table 1) [11].

In the autumn of the children’s first school year at age 7, the next follow-up was conducted. The follow-up was conducted by questionnaires for the parents (participation rate, 90%) and it included information on children’s growth, development and health, and social situation [13]. In the spring of the children’s first school year, the next follow-up was conducted. Two questionnaires were sent to parents: one questionnaire to themselves and another for the children’s teachers. Parents completed a modified (1 of the internalising items was modified) Rutter A2 [14] and teachers filled out a Rutter B2 [15] questionnaire on children’s psychomotor development and behaviour. The participation rate for the parents’ Rutter A2 was 90%, and for teachers, the participation rate for the Rutter B2 was 92% (Table 1) [13]. The Rutter scales screen children’s behavioural and emotional features [15].

The latest follow-up for the whole cohort was conducted at the age of 16 years. The follow-up consisted of a questionnaire (participation rate, 77.9%) and clinical examination (participation rate, 73.7%). The questionnaire included questions about participants’ family, school, physical health, physical activity, sexual behaviour, smoking, alcohol and use of other substances, nutrition, living habits, and hobbies. The questionnaire included several psychiatric screening tools: the 21-item PROD-screen for prodromal psychotic symptoms [16], the Youth Self Report for evaluating the competencies and problems of 11-year to 18-year-old adolescents [17], the Strengths and Weakness of Attention-Deficit/Hyperactivity Disorders Symptoms and Normal Behaviour Scale (SWAN) to detect attention deficit-hyperactivity disorder (ADHD) symptoms [18], and the Toronto Alexithymia Scale to measure alexithymia [19]. The clinical examination included, for example, weight, height, and waist-hip measurements, sitting height, spirometry, blood pressure, pulse rate, blood samples, physical activity tests, and prick tests (Table 1). In the 16-year follow-up, adolescents and their parents were requested for consent to use the collected data.

The follow-up of the NFBC 1986 contained multiple subsamples. One of the psychiatric subsamples was based on the SWAN screening in the 16-year follow-up, including 268 likely ADHD cases and 196 controls [20]. Participants were interviewed (ages 16-18 years) using the Schedule for Affective Disorders and Schizophrenia for School-Age Children-Present and Lifetime version [21]. The follow-up also included several intelligence and cognitive tests (more detailed information is presented in the Supplementary Material 1). Parents also completed a questionnaire concerning their own attention problems using the ADHD-Rating Scale-IV and background information (Table 2).

The next psychiatric subsample (Oulu Brain and Mind Study I) was conducted in 2007-2010 (participants 21-24 years old) [22]. In total 329 of the invited 763 members of the NFBC 1986 participated. The sample was based on participants at risk of developing psychosis (familial risk for psychosis: n= 78 and symptomatic risk for psychosis: n= 58), and the comparison group included subjects who had been treated for a psychotic episode (n= 27), those with ADHD (n= 52) and a random sample of the cohort (n= 80). Additionally, 34 individuals who had participated as controls in the earlier ADHD study participated in the Oulu Brain and Mind I study. Participants completed a questionnaire including questions on their background information, relationships, quality of life, substance use, and several psychiatric and psychological screening instruments (more detailed information is found in the Supplementary Material 1). The clinical examination included blood sampling (DNA from blood) and a urine sample (to detect use of drugs and medications). Psychiatric nurses also interviewed participants with questions about illness and treatments, the Structured Interview for Prodromal Syndromes [23], the ADHD questionnaire, and the Structured Clinical Interview for DSM-IV Axis I Disorders (SCID-I) [24]. Furthermore, the participants underwent structural magnetic resonance imaging (MRI), diffusion-tensor imaging, resting-state functional MRI, and functional MRI with 3 different tasks: the Sternberg verbal working memory task [25], the Human Causal Learning prediction error task [26], and a facial recognition task [27]. The MRI scans were performed with a 1.5 T scanner and the entire procedure took 60-75 minutes. The participants also received several cognitive tests, about which more detailed information is found in the Supplementary Material 1. Information about the results of the MRI scans was given to the participants (Table 2).

The next psychiatric sub-study (Oulu Brain and Mind Study II) was conducted at the age of 25-27 years [28]. The cases in the study were selected based on parental exposure to maternal cigarette smoking (n= 253), and the controls were matched by maternal education and geographical region (n = 218). The participants completed a questionnaire including information about their background factors, lifestyle, and several screening instruments (Supplementary Material 1). Structural MRI, diffusion-tensor imaging, magnetisation transfer imaging, and resting-state functional MRI were conducted. The MRI scanning was performed with a 1.5 T scanner and the entire procedure took 45-60 minutes. The participants were also assessed using several cognitive tests (Supplementary Material 1). Blood and urine samples were also taken. The interviews included, among other things, psychiatric SCID-I screen. The participants received information about the results of the MRI scans, cognitive tests, and possible mental disorders (Table 2).

The NFBC 1986 also includes other sub-studies. The first substudy was conducted among live-born preterm children with a birth weight < 1,750 g and their controls at the age of 8 years. The sub-study included a neurological examination and psychological assessments [29]. In the Oulu Back Study, a questionnaire including questions about low back pain history, background factors, leisure activities and history of injuries was sent to the cohort members living in Oulu and the surrounding municipalities at the age of 18 years [30]. The following sub-studies were’ conducted when the cohort members were 19-22 years and 29-32 years of age, with MRI scans of the lumbar spine [31]. A subpopulation of the NFBC at the age of 24-years took part in the Ester–Preterm Birth, Pregnancy and Offspring Health in Adult Life study. The study included blood sampling and measurements of blood pressure, body mass index, and waist and hip circumference [32]. At the age of 26 years, the gynaecological health of young female was studied, and a questionnaire on socio-demographic and other health background factors (mainly about reproduction, menstruation, and infertility) was sent to a subpopulation of the NFBC 1986 [33] (Supplementary Material 2).

### Final datasets of the Northern Finland Birth Cohort 1986 and the comparison birth cohort

From the NFBC 1986, we excluded those who did not survive the perinatal period (stillbirths: n= 47, 0.5%; those who died within the first 7 days of life: n= 36, 0.4%) in order to make the datasets comparable. After exclusion, 9,396 members of the NFBC 1986 (male: n= 4,839, 51.5%; female: n= 4,557, 48.5%) and 8,959 members from the comparison group (male: n= 4,550, 50.8%; female: n= 4,409, 49.2%) were included in all analyses.

### Mental disorders

The focus of this study was mental disorder diagnoses from the age of 2 years to 28 years. The data covered the period from 1987 to 2014 in the NFBC 1986 dataset and from 1989 to 2015 in the comparison dataset. Data on mental disorder diagnosis were obtained from the Care Register for Health Care (CRHC), which is maintained by the THL. In the present study, we analysed a broad range of International Statistical Classification of Diseases and Related Health Problems (ICD)-diagnosed psychiatric disorders classified into several classes (Supplementary Material 3). We used ICD-9 (from 1987 to 1995) and ICD-10 (from 1996 to 2015) classifications. The CRHC is one of the oldest individual-level hospital discharge registers and it has nationwide hospital discharge information on inpatient visits starting from 1967. From 1998 onwards, the register also includes information on specialised outpatient care. We used specialised outpatient care diagnoses from the age of 13 years to 28 years (in NFBC from 1998 and in the comparison cohort from 2000). By law, all hospitals in Finland are obligated to report all inpatient care. The outpatient care data cover public hospitals only. Several studies have indicated that the quality of CRHC is high [34].

### Suicidal behaviour

Suicidal behaviour was studied with 2 outcome variables: suicide attempts (including self-harm) and suicides from 2 years to 28 years of age. The CRHC was used to identify patients who had a diagnosis (primary diagnosis, secondary diagnosis, or external cause diagnosis) of suicide attempt, including self-harm or suicide attempt (Supplementary Material 3). Death by suicide was defined using the relevant statistical underlying cause-of-death diagnoses, obtained from the Cause of Death Registers at Statistics Finland (Supplementary Material 3).

### General characteristics

We used marital status and education as confounding factors. Information on marital status at the age of 28 years was obtained from the Central Population Register and was classified into 2 classes: married and single. Education indicated the highest achieved level of education at the age of 28 years (secondary education or less and tertiary education). Information on participants’ education was obtained from Statistics Finland.

### Statistical analysis

The prevalence of general characteristics was reported, and differences were estimated using the chi-square test. The cumulative incidence rates of mental disorders and suicidal behaviour at the ages of 2 years to 28 years were calculated for both cohorts (NFBC 1986 and the comparison cohort) and the significance of differences was estimated with the chi-square test. Risk ratios (RRs) with 95% confidence intervals (CIs) were calculated by sex separately in each diagnosis group where the number of cases was large enough. Additionally, the Mantel-Haenszel risk ratio (RR_MH_) was used to calculate the adjusted RRs (adjusted for general characteristics: education, and marital status) in each diagnosis group where the number of cases within each adjustor was large enough. The numbers of suicide attempts within each general characteristic were too low to be analysed with RR_MH_. The analysis was performed using R version 1.4.1106 (R Foundation for Statistical Computing, Vienna, Austria).

### Ethics statement

The NFBC 1986 is kept under review by the Ethical Committee of the Northern Ostrobothnia Hospital District. Register-based 1987 FBC obtained ethics approval from the Finnish Institute for Health and Welfare (ethical committee §28/2009).

## RESULTS

Table 3 summarises the general characteristics of the NFBC 1986 and comparison cohort by sex. More female participants of the NFBC 1986 were married than female participants of the comparison cohort (χ^2^= 5.49; p= 0.019).

In male, no significant differences in mental disorders were found between NFBC 1986 and the comparison cohort (Table 4). In female, a lower risk in NFBC 1986 was found in several diagnostic categories: any psychiatric or neurodevelopmental disorder (RR, 0.89; 95% CI, 0.81 to 0.98; p= 0.014); schizophrenia, schizotypal and delusional disorders (RR, 0.68; 95% CI, 0.49 to 0.93; p= 0.016); other non-affective psychosis (RR, 0.67; 95% CI, 0.48 to 0.92; p= 0.014); neurotic, stress-related, and somatoform disorders (RR, 0.78; 95% CI, 0.68 to 0.90; p< 0.001); anxiety disorder (RR, 0.79; 95% CI, 0.66 to 0.96; p= 0.015); post-traumatic stress disorder (RR, 0.60; 95% CI, 0.41 to 0.88; p= 0.008); and learning and coordination disorders (RR, 0.48; 95% CI, 0.31 to 0.77; p= 0.002) (Table 5). After adjusting the risk ratios for education and marital status, a lower risk was found for neurotic, stress-related, and somatoform disorders (RR_MH_, 0.80; 95% CI, 0.70 to 0.91; p= 0.001); anxiety disorder (RR_MH_, 0.81; 95% CI, 0.67 to 0.98; p=0.029); post-traumatic stress disorder (RR_MH_, 0.61; 95% CI, 0.42 to 0.90; p= 0.011); and learning and coordination disorders (RR_MH_, 0.52; 95% CI, 0.33 to 0.82; p= 0.004) (Table 5).

No significant differences were found in suicidal behaviour for males between NFBC 1986 and the comparison cohort. In female, there was a decreased risk for suicide attempts in the NFBC 1986 cohort (RR, 0.67; 95% CI, 0.49 to 0.92; p= 0.011). The numbers of suicides were too low to be analysed: 6 in the NFBC 1986 cohort (0.1%) and 5 in the FBC 1987 cohort (0.1%) (Table 6).

## DISCUSSION

The results did not support our first hypothesis of increased use of mental health services in the NFBC 1986. On the other hand, our second hypothesis gained some support, as female participants of the NFBC 1986 had a decreased risk for suicide attempts; however, this was not due to a higher number of participants receiving psychiatric treatment.

Participation in the NFBC 1986 did not increase the use of mental health services; instead, the opposite occurred in female. The female members of NFBC 1986 did seek help for mental health problems less than the comparison cohort and therefore had fewer psychiatric diagnoses in healthcare registers. The previous Finnish psychiatric UKKI study found an association between participation in a follow-up study and the use of mental healthcare services, as study participants used more psychiatric care services than the comparison group. The difference found in the UKKI study may have been due to psychiatric interviews conducted with the study participants [6].

Several studies have reported higher levels of mental healthcare utilisation among female [35]. Sex differences in the use of mental health services have been associated with sex-specific patterns in the pathology of mental disorders; female more frequently experience anxiety and depressive disorders, while male are more likely to suffer from impulsivity and addiction [36]. Sex differences in the use of mental healthcare have been explained in terms of differences in the need for care, as well as attitudinal differences, psychosocial factors, and associations with a number of socioeconomic and family-related determinants [37]. The above findings might be relevant to our findings. The follow-up of the NFBC 1986 might have affected female participants differently than male participants.

Computer-assisted and Internet-based treatments (e-interventions) for anxiety disorder and depression have grown over the past decades, and previous findings have substantiated the efficacy of e-interventions for adult and youth anxiety and depression [38]. Internet-based psychotherapy for anxiety disorder and depression has been proven to be clinically effective, with outcomes equivalent to those of face-to-face psychological therapy [38,39]. For example, the Internet-based myCompass program (a fully-automated, self-help, public health intervention) reduced symptoms for individuals with mild to moderate depression, anxiety and stress [40]. An effective treatment for anxiety disorder does not need to be long-term and intensive. Very light interventions have been found to be effective. This might be an explanation of the findings of the present study: the contact and questionnaires used in the NFBC 1986 might have helped some participants with mild anxiety and stress symptoms, as a lower risk for anxiety disorder and post-traumatic stress disorder was found in the NFBC 1986 cohort. A limitation of our study was that we had no data on the use of primary healthcare services, and thus on e-interventions.

Learning disorders are usually diagnosed in the early school years, and the DSM-IV describes learning disorders as occurring when an individual’s achievement on individually administered standardised tests is substantially below that expected for age, schooling, and level of intelligence [41]. In the NFBC 1986, the first follow-ups after the antenatal period were conducted in the children’s first school year: in the autumn, parents completed a questionnaire about children’s growth and development, and in the spring, parents and teachers evaluated the children’s behaviour with the Rutter scale.

It is rather difficult to explain the results for schizophrenia and other non-affective psychosis. In the NFBC 1986 cohort, the incidence of these disorders was significantly lower than in the FBC 1987 cohort. It is very unlikely that any light intervention would affect the incidence of severe psychiatric illnesses. Nevertheless, the finding for non-affective psychosis was not in line with our hypothesis.

Our second hypothesis was partly supported by the suicidal behaviour findings. Suicidal behaviour was lower in female NFBC 1986 members, but that was not due to a higher incidence of using mental health services. The effect of interventions on suicidal behaviour has been previously reported. The intervention does not need to be intensive to be effective; for example, media sources have an important degree of leverage in influencing the suicide rate by reporting suicides. An awareness program has also been reported to be successful in preventing suicide [42].

In conclusion, participation in the NFBC 1986 cohort study did not increase the use of mental health services; rather, the opposite occurred in females. The NFBC 1986 may not be completely representative on the population level in terms of psychiatric outcomes. The effects of participation in epidemiological followup studies have been under-examined earlier, and the results need to be replicated. The follow-up studies conducted among the participants might be considered as interventions.

### Strengths

To our knowledge, the effects of participation in a prospective epidemiological study have rarely been studied. The data used in this study were from Finnish registers, which have been found to be high-quality. The quality of the CRHC is high [34] and the coding of causes of death for mortality statistics is appropriate, with a sincere quality coding process [43]. The response rate in the NFBC 1986 cohort can be considered high, since poor response rates in follow-up cohort studies are causing increasing concern [44].

### Limitations

Even though the follow-up in the NFBC included many questionnaires and a clinical examination, only a few of the themes were focused on psychiatric measurements. Although the NFBC is a relatively large cohort, the number of suicides was too low for analysis. The prevalence rates were also in some diagnostic groups in both the NFBC 1986 and the comparison cohort, which could have led to false positives.

## Figures and Tables

**Table 1.** Follow-ups of the Northern Finland Birth Cohort 1986

Age (main reference)	Target population (n)	Questionnaire data (participation rate)	Psychiatric items in the questionnaires	Clinical examination (participation rate)
Antenatal period [11]	All births in the area (9,575)	Questionnaire on family’s background information for mothers n=9,479 (99%)	Maternal fatigue, alcohol use and smoking during pregnancy; Paternal alcohol use and smoking	NA
7 yr [13]	9,357	Questions on children’s growth, development and health, school and family type, and social situation for parents n=8,416 (90%)	NA	NA
8 yr [13]	9,357	n=8,525 (91.7%) assessed by teachers; n=8,370 (90.0%) assessed by parents	Teachers assessed the children’s behaviour with the Rutter B2 scale; Parents completed the modified Rutter A2 scale and questions on children’s psychomotor development and behaviour	NA
15-16 yr	9,340	Family, friends, school, health, behaviour, nutrition, physical activity, living habits and hobbies from adolescent n=7,182 (71.9%); Of adolescent’s health, development and behaviour from parents n=6,866 (75.5%)	PROD-screen for prodromal psychotic symptoms, the Youth Self Report, the Strengths and Weaknesses of Attention-Deficit/Hyperactivity Disorder Symptoms and Normal Behaviour Scale, Toronto Alexithymia Scale; Smoking and use of alcohol and other substances	Weight, height, waist-hip measurements, spirometry, blood pressure, pulse rate, blood samples., physical activity, prick tests, and questions on puberty, nutrition, and the use of intoxicants n=6,795 (73.5%)

NA, not applicable.

**Table 2.** Psychiatric sub-studies of the Northern Finland Birth Cohort 1986

Age		Target population	Questionnaire data (participation rate)	Psychiatric items in the questionnaires	Clinical examination (participation rate)
ADHD					
	16 yr [20]	A subsample based on SWAN-screen, cases n=487 and controls n=315	Cases: n=268 (55.0%), controls: n=196 (62.2%)	-	Psychiatric evaluation using the Schedule for Affective Disorders and Schizophrenia for School-Age Children-Present and Lifetime version, cognitive tests: WAIS-R Vocabulary and Block Design, the Stop-Signal, Attentional Network Task, the Conners Continuous Performance Test II, WAIS-III Processing Speed Index, the Wechsler Memory Scale, the Verbal Fluency Test and the Fingertip Tapping Test from the NEPSY, the Trail marking test; Blood samples
Oulu Brain and Mind					
	21-24 yr [22]	Cases at risk of developing psychosis n=389 and their controls; Psychotic episode n=78, ADHD n=103, random sample of the cohort n=193	Background information, relationships, quality of life, substance use, handedness, coping; Cases: n=136 (35.0%); Controls: psychotic episode n=27 (34.6%), ADHD n=52 (50.5%), random sample of the cohort n=80 (41.5%)	LOT, the Relationship Questionnaire, Sense of Coherence Scale, PROD-screen, Schizotypal Personality, ASR, Parental Bonding Instrument, Life Event Checklist, TADS, Magical Ideation Scale and Perceptual Aberration Scale	Cognitive tests: Vocabulary, Matrix Reasoning and Digit Span, California Verbal Learning Test - Research Edition, Logical Memory, Verbal fluency, Grooved Pegboard and CANTAB, tests of Paired Associates Learning, Spatial Working Memory, Stockings of Cambridge, Rapid Visual Information Processing and Information Sampling Test; Blood and urine sampling; Structural MRI, DTI, resting-state functional MRI, fMRI; Structured Interview for Prodromal Syndromes, ADHD interview and the SCID-I
	25-27 yr [28]	Cases based on exposure to maternal cigarette smoking (n=698) and their controls (n=698)	Background and lifestyle information, sleep questions, smoking, substance use and gambling, handedness, physical activity, diet, health, LOT; Cases: n=218 (31.0%); Controls: n=253 (36.0%)	TADS, LOT, ASR, PROD-screen, Personality Inventory NEO-PI, Temperament and Character Inventory	Interview including SCID-I, family history of psychiatric disorders, psychiatric treatment history, Brief Nicotine Dependence Interview; Cognitive tests: Weichel’s Adult Intelligence Scale, PAL, Semantic Fluency, the Grooved Pegboard test, the Stroop test, modified Stop Signal Test from CANTAB; MRI of the brain (T2, DTI, MTR, R-fMRI); Blood and urine samples

SWAN, Strengths and Weakness of Attention-Deficit/Hyperactivity Disorders Symptoms and Normal Behaviour Scale; ADHD, attention deficit–hyperactivity disorder; LOT, Life Orientation Test; ASR, Adult Self Report; TADS, Trauma and Distress Scale; NEO-PI, NEO Personality Inventory; WAIS-R, Wechsler Adult Intelligence Scale; NEPSY, A Developmental Neuropsychological Assessment; CANTAB, Cambridge Neuropsychological Test Automated Battery; MRI, magnetic resonance imaging; DTI, diffusion-tensor imaging; fMRI, functional magnetic resonance imaging; SCID-I, Structured Clinical Interview for DSM-IV Axis I Disorders; PAL, paired associates learning; T2, transverse relaxation time; MTR, magnetization transfer ratio.

**Table 3.** General characteristics of the Northern Finland Birth Cohort 1986 (NFBC 1986) and the comparison cohort by sex

Characteristics		Male		χ^2^ (p-value)	Female		χ^2^ (p-value)
		NFBC 1986 (n=4,839)	Comparison cohort (n=4,550)		NFBC 1986 (n=4,557)	Comparison cohort (n=4,409)	
Education				0.14 (0.710)			3.51 (0.061)
	Secondary education or less	3,470 (71.7)	2,706 (71.3)		2,483 (55.5)	2,490 (56.5)	
	Tertiary education	1,369 (28.3)	1,304 (28.7)		2,074 (45.5)	1,919 (43.5)	
Marital status				0.00 (>0.999)			5.49 (0.019)
	Single	3,858 (79.7)	3,628 (79.7)		3,121 (68.5)	3,121 (70.8)	
	Married	981 (20.3)	922 (20.3)		1,436 (31.5)	1,288 (29.2)	

Values are presented as number (%).

**Table 4.** Cumulative incidence and comparative risk of psychiatric disorders in male aged 2-28 years

Diagnostic group		NFBC 1986, n (%)	Comparison cohort, n (%)	χ^2^	p-value	RR (95% CI)	p-value	RR_MH_ (95% CI)^1^	p-value
Any psychiatric or neurodevelopmental disorder		691 (14.3)	625 (13.7)	0.54	0.461	1.04 (0.94, 1.15)	0.443	1.04 (0.94, 1.14)	0.489
Organic mental disorders		14 (0.3)	3 (0.1)	NA	NA	NA	NA	NA	NA
Mental disorders due to psychoactive substance use		206 (4.3)	181 (4.0)	0.33	0.563	1.06 (0.88, 1.30)	0.529	NA	NA
	Alcohol	164 (3.4)	145 (3.2)	0.24	0.623	1.06 (0.85, 1.32)	0.583	NA	NA
	Other substances	90 (1.9)	72 (1.6)	0.91	0.341	1.18 (0.86, 1.60)	0.302	NA	NA
Schizophrenia, schizotypal, and delusional disorders		91 (1.9)	79 (1.7)	0.27	0.655	1.08 (0.80, 1.46)	0.600	1.08 (0.80, 1.45)	0.624
	Schizophrenia	34 (0.7)	28 (0.6)	0.16	0.693	1.14 (0.69, 1.88)	0.602	NA	NA
	Other non-affective psychosis	86 (1.8)	76 (1.7)	0.10	0.750	1.06 (0.78, 1.44)	0.691	1.06 (0.78, 1.44)	0.717
Mood disorders		248 (5.1)	241 (5.3)	0.11	0.743	0.97 (0.81, 1.15)	0.708	0.96 (0.81, 1.15)	0.673
	Mania and bipolar	21 (0.4)	26 (0.6)	0.63	0.426	0.76 (0.43, 1.35)	0.346	NA	NA
	Depression	234 (4.8)	225 (4.9)	0.04	0.843	0.98 (0.82, 1.17)	0.806	0.97 (0.82, 1.16)	0.771
Neurotic, stress-related, and somatoform disorders		249 (5.1)	223 (4.9)	0.24	0.621	1.05 (0.88, 1.25)	0.588	1.04 (0.87, 1.24)	0.652
	Obsessive-compulsive disorder	18 (0.4)	16 (0.4)	0.00	>0.999	1.06 (0.54, 2.07)	0.870	NA	NA
	Anxiety disorders	141 (2.9)	125 (2.7)	0.18	0.672	1.06 (0.84, 1.34)	0.627	1.06 (0.83, 1.34)	0.655
	Post-traumatic stress disorder	13 (0.3)	12 (0.3)	0.00	>0.999	1.02 (0.47, 2.23)	0.963	NA	NA
Behavioural syndromes associated with eating, sleep, or puerperium		37 (0.8)	32 (0.7)	0.01	0.912	1.06 (0.66, 1.70)	0.816	1.08 (0.68, 1.74)	0.734
	Eating disorders	6 (0.1)	4 (0.1)	NA	NA	NA	NA	NA	NA
Disorders of adult personality		55 (1.1)	51 (1.1)	0.00	>0.999	1.01 (0.69, 1.48)	0.943	NA	NA
	Emotionally unstable personality	19 (0.4)	16 (0.4)	0.02	0.876	1.12 (0.57, 2.17)	0.745	NA	NA
Disorders diagnosed in childhood or in adolescence		246 (5.0)	266 (5.8)	2.50	0.114	0.87 (0.73, 1.03)	0.104	0.87 (0.73, 1.02)	0.092
	Autism spectrum disorders	23 (0.5)	26 (0.6)	0.25	0.615	0.83 (0.48, 1.46)	0.518	NA	NA
	Learning and coordination disorders	110 (2.3)	115 (2.5)	0.64	0.422	0.89 (0.68, 1.16)	0.384	0.89 (0.69, 1.16)	0.397
	ADHD	38 (0.8)	51 (1.1)	2.47	0.116	0.70 (0.46, 1.06)	0.094	NA	NA
	Conduct and oppositional disorders	58 (1.2)	67 (1.5)	1.14	0.286	0.81 (0.57, 1.15)	0.247	NA	NA

NFBC 1986, Northern Finland Birth Cohort 1986; RR, crude risk ratio; RR_MH_, Mantel-Haenszel risk ratio; CI, confidence interval; ADHD, attention deficit–hyperactivity disorder; NA, not applicable.

1 Adjusted for marital status and education.

**Table 5.** Cumulative incidence and comparative risk of psychiatric disorders in female aged 2-28 years

Diagnostic group		NFBC 1986, n (%)	Comparison cohort, n (%)	χ^2^	p-value	RR (95% CI)	p-value	RR_MH_ (95% CI)^1^	p-value
Any psychiatric or neurodevelopmental disorder		734 (16.1)	796 (18.0)	5.87	0.015	0.89 (0.81, 0.98)	0.014	0.91 (0.83, 1.00)	0.056
Organic mental disorders		7 (0.2)	3 (0.1)	NA	NA	NA	NA	NA	NA
Mental disorders due to psychoactive substance use		124 (2.7)	129 (2.9)	0.27	0.602	0.93 (0.73, 1.19)	0.558	0.96 (0.76, 1.23)	0.753
	Alcohol	96 (2.1)	101 (2.3)	0.27	0.601	0.92 (0.70, 1.21)	0.552	0.95 (0.72, 1.25)	0.705
	Other substances	44 (1.0)	44 (1.0)	0.00	0.961	0.97 (0.64, 1.47)	0.876	NA	NA
Schizophrenia, schizotypal, and delusional disorders		63 (1.4)	90 (2.0)	5.41	0.020	0.68 (0.49, 0.93)	0.016	NA	NA
	Schizophrenia	18 (0.4)	27 (0.6)	1.71	0.191	0.65 (0.36, 1.17)	0.145	NA	NA
	Other non-affective psychosis	60 (1.3)	87 (2.0)	5.59	0.018	0.67 (0.48, 0.92)	0.014	NA	NA
Mood disorders		416 (9.1)	450 (10.2)	2.62	0.105	0.90 (0.79, 1.02)	0.098	0.92 (0.81, 1.04)	0.175
	Mania and bipolar	41 (0.9)	38 (0.9)	0.01	0.937	1.04 (0.67, 1.62)	0.848	NA	NA
	Depression	398 (8.7)	428 (9.7)	2.20	0.138	0.90 (0.79, 1.03)	0.128	0.92 (0.81, 1.05)	0.214
Neurotic, stress-related and somatoform disorders		344 (7.5)	425 (9.6)	12.22	<0.001	0.78 (0.68, 0.90)	<0.001	0.80 (0.70, 0.91)	0.001
	Obsessive-compulsive disorder	31 (0.7)	36 (0.8)	0.39	0.531	0.83 (0.52, 1.34)	0.454	0.86 (0.53, 1.38)	0.520
	Anxiety disorders	191 (4.2)	233 (5.3)	5.70	0.017	0.79 (0.66, 0.96)	0.015	0.81 (0.67, 0.98)	0.029
	Post-traumatic stress disorder	42 (0.9)	68 (1.5)	6.62	0.010	0.60 (0.41, 0.88)	0.008	0.61 (0.42, 0.90)	0.011
Behavioural syndromes associated with eating, sleep, or puerperium		79 (1.7)	101 (2.3)	3.29	0.070	0.75 (0.56, 1.01)	0.059	0.77 (0.58, 1.03)	0.078
	Eating disorders	61 (1.3)	77 (1.7)	2.47	0.116	0.75 (0.54, 1.05)	0.097	0.78 (0.56, 1.09)	0.141
	Disorders of adult personality	79 (1.7)	92 (2.1)	1.31	0.252	0.83 (0.62, 1.12)	0.222	0.86 (0.64, 1.16)	0.330
	Emotionally unstable personality	52 (1.1)	59 (1.3)	0.56	0.454	0.85 (0.59, 1.24)	0.399	NA	NA
Disorders diagnosed in childhood or in adolescence		161 (3.5)	189 (4.3)	3.20	0.074	0.82 (0.67, 1.01)	0.065	0.85 (0.69, 1.04)	0.122
	Autism spectrum disorders	9 (0.2)	7 (0.2)	NA		NA	NA	NA	NA
	Learning and coordination disorders	28 (0.6)	54 (1.2)	9.31	0.002	0.48 (0.31, 0.77)	0.002	0.52 (0.33, 0.82)	0.004
	ADHD	9 (0.2)	14 (0.3)	NA		NA	NA	NA	NA
	Conduct and oppositional disorders	62 (1.4)	54 (1.2)	0.23	0.635	1.11 (0.77, 1.60)	0.570	NA	NA

NFBC 1986, Northern Finland Birth Cohort 1986; RR, crude risk ratio; RR_MH_, Mantel-Haenszel risk ratio; CI, confidence interval; ADHD, attention deficit–hyperactivity disorder; NA. not applicable.

1 Adjusted for marital status and education.

**Table 6.** Cumulative incidence and comparative risk for suicidal behaviour in participants aged 2-28 years

Sex		NFBC 1986, n (%)	Comparison cohort, n (%)	χ^2^	p-value	RR (95% CI)	p-value
Male							
	Suicide attempt	70 (1.4)	83 (1.8)	1.86	0.173	0.79 (0.58, 1.09)	0.148
	Suicide	29 (0.6)	22 (0.5)	0.39	0.534	1.24 (0.71, 2.15)	0.446
Female							
	Suicide attempt	65 (1.4)	94 (2.1)	6.01	0.014	0.67 (0.49, 0.92)	0.011
	Suicide	6 (0.1)	5 (0.1)	NA	NA	NA	NA

NFBC 1986, Northern Finland Birth Cohort 1986; RR, risk ratio: CI, confidence interval; NA, not applicable.

## SUPPLEMENTARY MATERIALS

Supplementary Material 1.

Supplementary Material 2. Other sub-studies of the NFBC 1986

Supplementary Material 3. Psychiatric diagnostic groups with respective codes according to ICD-9 (1987-1995), and ICD-10 (1996-) classifications.
