# Supplementary material for: Association of participation in the Northern Finland Birth Cohort 1986 with mental disorders and suicidal behaviour
Source: Epidemiol Health. 2022 Jan 3;44:e2022005. doi: 10.4178/epih.e2022005 (PMC9016388; doi:10.4178/epih.e2022005)
Supplement: Supplementary Material 1. [file epih-44-e2022005-suppl1.docx]

**Supplementary Material 1.**

**Cognitive tests on the subsample at 16-year-old**

WAIS-R Vocabulary and Block Design [1] Go reaction time Go-RT and SD of the reaction time from The Stop-Signal task [2], Attentional Network Task ANT [3], The Conners Continuous Performance Test II CPT [4], WAIS-III Processing Speed Index, Spatial Span and Letter-Number Sequences from the Wechsler Memory Scale [1], The Verbal Fluency Test and the Fingertip Tapping Test from the NEPSY [5], and the Trail marking test [6].

**Screening instruments on the Oulu Brain and Mind I study**

Life orientation test LOT-R [7], Handedness, Sense of Coherence Scale SOC-13 [8], PROD-screen [9], Schizotypal Personality Questionnaire SPQ-B [10], The Relationship Questionnaire RQ [11], Adult Self Report ASR [12], Parental Bonding Instrument PBI [13], Life Event Checklist LEC [14], Trauma and Distress Scale TADS [15], Magical Ideation Scale MIS [16] and Perceptual Aberration Scale PER [17].

**Cognitive tests on the Oulu Brain and Mind I study**

Vocabulary, Matrix Reasoning and Digit Span WAIS III [18], California Verbal Learning Test - Research Edition (CVLT) [19], Logical Memory WMS-R [1], Verbal fluency, Grooved Pegboard and Cambridge Neuropsychological Test Automated Battery CANTAB tests of Paired Associates Learning PAL [20], Spatial Working Memory SWM, Stockings of Cambridge SOC, Rapid Visual Information Processing RVP and Information Sampling Test.

**Screening instruments on the Oulu Brain and Mind II study**

TADS, Life Orientation Test LOT, NEO Personality Inventory NEO-PI [21], Temperament and Character Inventory TCI [22], participated in an interview including SCID I, Family history of psychiatric disorders, Psychiatric treatment history and Brief Nicotine Dependence Interview SSAGA [23].

**Cognitive tests on the Oulu Brain and Mind II study**

Weichel’s Adult Intelligence Scale WAIS-3 [24], PAL [20], Semantic Fluency [25], the Grooved Pegboard test using dominant hand [26], the Stroop test [27], modified Stop Signal Test MSST from CANTAB.

**References**

[1]Wechsler D. Wechsler memory scale-revised. Psychological Corporation 1987.

[2]Logan GD, Schachar RJ, Tannock R. Impulsivity and Inhibitory Control. Psychol Sci 1997;8(1):60-64.

[3]Fan J, McCandliss BD, Sommer T, Raz A, Posner MI. Testing the efficiency and independence of attentional networks. J Cogn Neurosci 2002;14(3):340-347.

[4]Conners CK, Staff M, Connelly V, Campbell S, MacLean M, Barnes J. Conners’ continuous performance Test II (CPT II v. 5). Multi-Health Syst Inc 2000;29:175-196.

[5]Korkman M. NEPSY. A developmental neurop-sychological assessment. Test materials and manual 1998.

[6]Reitan RM. Manual for administration of neuropsychological test batteries for adults and children. : Neuropsychology Laboratory, Indiana University medical Center; 1959.

[7]Scheier MF, Carver CS, Bridges MW. Distinguishing optimism from neuroticism (and trait anxiety, self-mastery, and self-esteem): a reevaluation of the Life Orientation Test. J Pers Soc Psychol 1994 Dec;67(6):1063-1078.

[8]Antonovsky A. Unraveling the mystery of health: How people manage stress and stay well. San Francisco, CA, US: Jossey-Bass; 1987.

[9]Heinimaa M, Salokangas RK, Ristkari T, Plathin M, Huttunen J, Ilonen T, et al. PROD-screen--a screen for prodromal symptoms of psychosis. Int J Methods Psychiatr Res 2003;12(2):92-104.

[10]Meehl PE. Schizotaxia, schizotypy, schizophrenia. Am Psychol 1962;17(12):827-838.

[11]Bartholomew K, Horowitz LM. Attachment styles among young adults: A test of a four-category model. J Pers Soc Psychol 1991;61(2):226-244.

[12]Achenbach T, Rescorla LA. Manual for the ASEBA adult forms & profiles. Burlington, VT: University of Vermont, Research Center for Children, Youth, and Families; 2003.

[13]Parker G, Tupling H, Brown LB. A Parental Bonding Instrument. Br J Med Psychol 1979;52(1):1-10.

[14]Johnson J, McCutcheon S. Assessing life stress in older children and adolescents: Preliminary findings with the life events checklist. Washington, DC: Hemisphere; 1980.

[15]Patterson P, Skeate A, Schultze-Lutter F, Graf von Reventlow H, Wieneke A, Ruhrmann S, et al. The trauma and distress scale. Birmingham, UK: University of Birmingham 2002.

[16]Eckblad M, Chapman LJ. Magical ideation as an indicator of schizotypy. J Consult Clin Psychol 1983 Apr;51(2):215-225.

[17]Chapman LJ, Chapman JP, Raulin ML. Body-image aberration in schizophrenia. J Abnorm Psychol 1978;87(4):399-407.

[18]Wechsler D. WAIS-III administration and scoring manual. San Antonio, Texas: The Psychological Corporation; 1997.

[19]Delis DC, Kramer JH, Kaplan E, Ober BA. California verbal learning test research edition manual. San Antonio: The Psychological Corporation 1987.

[20]Sahakian BJ, Morris RG, Evenden JL, Heald A, Levy R, Philpot M, et al. A comparative study of visuospatial memory and learning in Alzheimer-type dementia and Parkinson's disease. Brain 1988 Jun;111 ( Pt 3):695-718.

[21] Costa PT, McCrae RR. The NEO personality inventory. Psychological assessment resources Odessa, FL; 1985.

[22]Cloninger CR, Przybeck TR, Svrakic DM, Wetzel RD. The Temperament and Character Inventory (TCI): A guide to its development and use. 1994.

[23]Bucholz KK, Cadoret R, Cloninger CR, Dinwiddie SH, Hesselbrock VM, Nurnberger JI, et al. A new, semi-structured psychiatric interview for use in genetic linkage studies: A report on the reliability of the SSAGA. J Stud Alcohol 1994;55(2):149-158.

[24]Wechsler D. Wechsler Adult Intelligence Scale. San Antonio, Texas: Psychological Corporation; 1997.

[25]Benton AL, Hamsher K. Multilingual Aphasia Examination. Iowa City, Iowa: University of Iowa; 1976.

[26]Trites R. Grooved Pegboard Instruction Manual. Lafayette: Lafayette Instrument; 1989.

[27]Strauss E, Sherman EMS, Spreen O. A compendium of neuropsychological tests: Administration, norms, and commentary (3rd ed.). Oxford University Press.; 2006.
