# Supplementary material for: Association of participation in the Northern Finland Birth Cohort 1986 with mental disorders and suicidal behaviour
Source: Epidemiol Health. 2022 Jan 3;44:e2022005. doi: 10.4178/epih.e2022005 (PMC9016388; doi:10.4178/epih.e2022005)
Supplement: Supplementary Material 2. — Other sub-studies of the NFBC 1986 [file epih-44-e2022005-suppl2.docx]

**Supplementary Material 2. Other sub-studies of the NFBC 1986**

| Age | Target Population  N | Questionnaire data  (Participation rate) | Psychiatric items in the Questionnaires | Clinical Examination  (Participation rate) |
| --- | --- | --- | --- | --- |
| Oulu Back Study | | | | |
| 18-years | Cohort members living in the Oulu and surrounding municipalities N=2969 | Questions of history of low back pain history, medical history, quality of life, nutrition, socioeconomic status, leisure activities, history of injuries, occupational exposure, sports activities, and psychological factors (N=2012, 68%) | Questions of smoking, anxiety and worrying | N/A |
| 19-22 years | Subsample of the cohort members living in the Oulu and surrounding municipalities N=874 | N/A | N/A | MRI-scan of the lumbar spine (N=558, 64%) |
| 29-32 years | Subsample of those who attended sub-study of 19-22 years N=558 | N/A | N/A | MRI-scan of the lumbar spine (N=375, 67%) |
| Gynaecological health of young women | | | | |
| 26 years | Gynaecological health of young women N=4503 and their mothers | Questions on socio-demographic and other health background factors  mainly about reproduction, menstruation, and infertility (N=2770, 50%) | N/A | N/A |
| Ester – Preterm Birth, Pregnancy and Offspring Health in Adult Life | | | | |
| 24-years | Preterm born subjects N=408 and controls N=579 | N/A | N/A | Blood samplings, Blood pressure, BMI, waist and hip measurements (N=378, 38.3%) |
| Preterm children | | | | |
| 8-years | Live-born preterm children with a birth weight <1750g (N=55) and controls (N=43) | N/A | N/A | A neurological examination and psychological assessments for cases N= 42 (75%) and controls N=43, MRI for all cases |
