# Supplementary material for: Association of participation in the Northern Finland Birth Cohort 1986 with mental disorders and suicidal behaviour
Source: Epidemiol Health. 2022 Jan 3;44:e2022005. doi: 10.4178/epih.e2022005 (PMC9016388; doi:10.4178/epih.e2022005)
Supplement: Supplementary Material 3. — Psychiatric diagnostic groups with respective codes according to ICD-9 (1987-1995), and ICD-10 (1996-) classifications. [file epih-44-e2022005-suppl3.docx]

**Supplementary Material 3. Psychiatric diagnostic groups with respective codes according to ICD-9 (1987-1995), and ICD-10 (1996-) classifications.**

| Diagnostic group | ICD-9 | ICD-10 |
| --- | --- | --- |
| **Any psychiatric or neurodevelopmental disorder** | 290-301, 303-316 | F0-F5, F60-F63, F68-F69, F8-F9 |
| **Organic mental disorders** | 290, 293, 294, 310 | F0 |
| **Mental disorders due to psychoactive substance use** | 291, 292, 303-305 | F1 |
| Alcohol | 291, 303, 3050A | F10 |
| Other substances | 292, 304, 3051-3059 | F11-F19 |
| **Schizophrenia, schizotypal and delusional disorders** | 295, 297, 2988A, 2989X, 3012C | F2 |
| Schizophrenia | 295 | F20, F25 |
| Other non-affective psychosis | 297, 2988A, 2989X, 3012C | F21- F24, F28, F29 |
| **Mood disorders** | 296, 3004A | F3 |
| Mania and bipolar | 2962-2964, 2967A | F30, F31 |
| Depression | 2961, 2968A, 3004A | F32-F34 |
| **Neurotic, stress-related and somatoform disorders** | 3000-3003, 3006-3009, 3078A, 3090A, 3092C-E, 3098A, 3098X, 3099X | F4 |
| Obsessive-compulsive disorder | 3003A | F42 |
| Anxiety disorders | 3000A, 3000B, 3000C, 3002B, 3002C, 3002D, 3002X | F400-F402, F408-F411, F413, F418, F419 |
| Post-traumatic stress disorder | 3098X | F431 |
| **Behavioral syndromes associated with eating, sleep or puerperium** | 3071A, 3074A, 3074F-H, 3075A-C, 3075E | F50, F51, F53 |
| Eating disorders | 3071A, 3075A-C, 3075E | F50 |
| **Disorders of adult personality** | 3010A, 3011D, 3012A, 3014A, 3015A-B, 3016A, 3017A, 3018B-X | F60, F61, F62 |
| Emotionally unstable personality | 3018D | F603 |
| **Disorders diagnosed in childhood or in adolescence** | 299, 313-315, 3120A, 3123C-D, 3070A-B, 3072A-D, 3073A, 3075D, 3076A-C, 3077A, 3092A-B, 3093A, 3094A | F80-F84, F88-F95, F98 |
| Autism spectrum disorders | 299 | F840, F845, F848, F849 |
| Learning and coordination disorders | 315 | F80-F83 |
| ADHD | 314 | F900 |
| Conduct and oppositional disorders | 3120A, 3123C-D, 3138A | F901, F910-F913, F918-F920, F928, F929 |
| **Suicide attempt** | E950A-E959X | X60-X84, Z91.5, Z72.8, Y87.0 |
| **Death by suicide** | E950-E959B, E959X, E950A-K, E951-E959C, E959X | X60-X84, Y870 |
